# Survival after cardiac arrest: what is the situation in Lithuania?

**DOI:** 10.1186/cc9720

**Published:** 2011-03-11

**Authors:** A Macas, G Baksyte, L Pieteris, A Vilke, A Peckauskas

**Affiliations:** 1 Lithuanian University of Health Sciences, Kaunas, Lithuania

## Introduction

Treatment of patients after sudden cardiac arrest remains a significant problem. Even after successful resuscitation, most patients have complications - one of the most serious and, unfortunately, very common being postanoxic brain injury. Aims of the study were to estimate the survival time for patients who had sinus rhythm restored after cardiac arrest but had neurological deficiency, and to estimate basic pathology that triggers cardiac arrest.

## Methods

Retrospective data analysis was performed in the coronary care unit of Lithuanian University of Health Sciences Hospital - Kaunas Clinics. Records of 56 patients were analysed (37.5% women and 62.5% men). Age ranged from 46 to 88 years. Average age was 65.32 ± 12.59. Sinus rhythm was restored for all patients after cardiac arrest, but had a neurological deficiency.

## Results

A total 89.28% of patients suffered out-of-hospital cardiac arrest. For 28.6% of patients it was enough to make CPR less than 15 minutes, before revival of sinus rhythm; 33.9% needed 15 to 30 minutes and 37.5% patients had to be resuscitated for more than 30 minutes. Almost one-half of patients (46.4%) did not survive 24 hours after resuscitation. The dominating basic pathology was acute myocardial infarction of the anterior wall (53.6%). The most common neurological deficiency was postanoxic coma (83.9%).

## Conclusions

Almost one-half of patients, which had revival of sinus rhythm after cardiac arrest and had neurological deficiency, did not survive 24 hours after resuscitation. The most common basic pathology, which caused cardiac arrest, was acute myocardial infarction with dominating anterior wall infarction.
